# 2025 Advances in Learning Health System Sciences (AiLHSS) Conference: Abstracts

**DOI:** 10.1002/lrh2.70066

**Published:** 2026-03-09

1

### 1.1 Genevieve B. Melton^1,2,3^; Amanda Trofholz^1^; Andy Oien^1^; Lindsay Bork Nichols^1^; Cory Schaffhausen^4,5^; Timothy Beebe^1,6^

#### 1.1.1 ^1^Center for Learning Health System Sciences, University of Minnesota, Twin Cities, Minneapolis, MN, USA; ^2^Department of Surgery, University of Minnesota, Twin Cities, Minneapolis, MN USA; ^3^Institute for Health Informatics, University of Minnesota, Twin Cities, Minneapolis, MN USA; ^4^Department of Medicine, University of Minnesota, Twin Cities, Minneapolis, MN USA; ^5^Hennepin Healthcare Research Institute, Minneapolis, MN USA; ^6^School of Public Health, University of Minnesota, Twin Cities, Minneapolis, MN USA

**Corresponding Author:** Amanda Trofholz, trofh002@umn.edu.

## 2 CONFERENCE SUMMARY

The Center for Learning Health System Sciences (CLHSS) at the University of Minnesota (UMN) hosted its third annual *Advances in Learning Health System Sciences* (AiLHSS) Conference on September 22‐23 at McNamara Alumni Center in Minneapolis, MN. This year's conference theme, “Building Bridges in LHS”, highlights AiLHSS’ role in both advancing the field of learning health systems while also providing networking and collaboration opportunities.

Reflecting a growing interest in LHS work, this year's conference had a 58% increase in registration with 346 registrants, including public health professionals, researchers, clinicians, hospital administrators, and industry partners. Day 1 included three keynote speakers, a poster session with over 60 presenters, plenary sessions featuring local, regional, and national research, and breakout sessions. The Day 1 agenda purposely included time for networking, including extended poster session times and an evening reception. Day 2 featured half‐day supplemental workshops.

Day 1 began with opening remarks from CLHSS Director Genevieve Melton‐Meaux, MD, PhD, and Shashank Priya, PhD, Vice President for Research & Innovation at UMN. The morning included a keynote address by Amy Kilbourne, PhD, MPH (US Department of Veterans Affairs and the University of Michigan), who highlighted how implementation science (IS) can align research translation with public health goals. Following was a plenary session showcasing regional and national initiatives in advancing IS in health care. Topics included reducing racial disparities in cardiac rehabilitation, avoiding unnecessary emergency department admissions for seniors, and improving guideline adherence for children with cerebral palsy. An extended poster session closed the morning agenda, offering additional networking opportunities.

The midday agenda shifted to pragmatic trials. Over lunch, Areef Ishani, MD, MS (Minneapolis VA and the UMN Department of Medicine) delivered a keynote focused on using pragmatic trials to produce rigorous evidence. A plenary session followed featuring LHS partnerships with public health, including presentations on statewide public health tools, case studies of partnerships between public health and health care, and example comparisons of Electronic Health Record (EHR) data and local public health data to better understand community health trends. Following this were two concurrent breakout sessions: (1) *Digital Transformation in Healthcare: Where Strategy, AI, and Investment Meet*, which explored how emerging technologies and venture investment are connecting to drive innovation across health care systems, and (2) *Data Action: Stories from the MN EHR Consortium*, which highlighted how Minnesota's EHR data is being utilized to provide insights into community health.

The end of the first day's agenda centered on the role data plays in improving healthcare practice. Six presenters participated in five‐minute Ignite session talks. Presentations highlighted innovative uses of data to improve health care, including designing systems to mitigate alarm fatigue, tracking substance‐use hospital visits after incarceration, and harmonizing nursing flowsheet data. Following was a closing keynote by Karandeep Singh, MD (University of California San Diego), who spoke on using AI to modernize healthcare delivery. The day ended with closing remarks by CLHSS Deputy Director Timothy Beebe, PhD, and a reception.

Day 2 of the conference featured two supplemental half‐day workshops. The first was an interactive IS workshop taught by Amy Kilbourne and local IS experts from UMN and the Minneapolis VA, which provided clinicians, researchers, and healthcare professionals with practical strategies to transform evidence‐based practices into IS studies. The second brought together members and partners of the Minnesota EHR Consortium to strengthen collaborations and explore innovative uses of the common data model.

This article features abstracts from selected plenary sessions and the “People's Choice” poster award winner. These abstracts showcase the innovation driving the future of learning health systems, highlighting how data, collaboration, approach, and evidence can be utilized to improve health and healthcare delivery. Collectively, they reflect the commitment of the LHS community to build more responsive, equitable, and effective health systems.

## 3 SUBSTANCE USE‐RELATED EMERGENCY DEPARTMENT VISITS IN THE TWIN CITIES METRO AMONG PEOPLE WITH RECENT INCARCERATION, 2016‐2025

### 3.1 Benjamin Bovell‐Ammon, MD, MPH^1^; Kriya Patel, MD^2^; Renee Van Siclen, MPP^3^; Wendy A. Miller, MD, MS^4^; Tyler N.A. Winkelman, MD, MSc^3,4^; On behalf of the MN EHR Consortium

#### 3.1.1 ^1^ Departments of Medicine and Healthcare Delivery and Population Sciences, UMass Chan Medical School‐Baystate and Baystate Medical Center, Springfield, MA, USA; ^2^ Section of General Internal Medicine, University of Chicago, Chicago, IL, USA; ^3^ Health, Homelessness, and Criminal Justice Lab, Hennepin Healthcare Research Institute, Minneapolis, MN, USA; ^4^ Division of General Internal Medicine, Department of Medicine, Hennepin Healthcare, Minneapolis, MN, USA

**Corresponding Author:** Benjamin Bovell‐Ammon ben.bovellammon@baystatehealth.org

**Introduction:** People with prior incarceration in a prison or jail have high rates of substance use disorders, overdoses, and other poor outcomes, including emergency department (ED) visits and hospitalizations. Prior research has characterized the involvement of opioids and other substances in fatal overdoses after release from incarceration, but less is known about their role in ED utilization and how that differs by incarceration history.

**Methods:** We used electronic health record (EHR) data from the Minnesota EHR Consortium (MNEHRC) on all visits to participating EDs in the seven counties that comprise the greater Minneapolis/St. Paul metropolitan area from January 2016 to March 2025. MNEHRC sites use a privacy‐preserving hashing algorithm to link individual EHR data to publicly available prison and jail records from the Minnesota Department of Corrections to identify ED visits by individuals with jail or prison incarceration within the prior 90 days, i.e., recent incarceration. We analyzed monthly counts of total and substance use‐related ED visits by incarceration status to characterize temporal trends and the associations between incarceration history, substance use, and ED utilization.

**Results:** We analyzed 10,949,040 ED visits, of which 208,439 (1.9%) were by a patient with recent incarceration. The overwhelming majority (93.0%) of those with recent incarceration had only jail incarceration, while 4.9% only had recent prison incarceration and 2.1% had both jail and prison incarceration. Compared to all ED visits, people with recent incarceration made up greater shares of substance use‐related ED visits (8.3%), opioid use‐related ED visits (9.5%) and, especially, nonfatal opioid overdose ED visits (17.0%). Annual trends showed that the share of all ED visits with recent incarceration decreased gradually from 2.1% in 2016 to 1.8% in 2024, the share of opioid use‐related ED visits with recent incarceration decreased gradually from 9.9% in 2016 to 8.8% in 2024, and the share of nonfatal opioid overdose ED visits with recent incarceration increased from 16.7% in 2016 to 20.8% in 2019 and then decreased to 14.9% in 2024. Among substance‐related ED visits, the frequency of opioids was similar between those with vs. without recent incarceration (24.4% vs. 20.8% of substance‐related visits); meanwhile, stimulants were more frequent (26.1% vs. 11.1% of substance‐related visits) and alcohol was less frequent (56.5% vs. 64.7% of substance‐related visits).

**Conclusions:** People with recent incarceration within the prior 90 days accounted for nearly 1 in 10 opioid use‐related ED visits and 1 in 6 nonfatal opioid overdose ED visits. Opioid related visits following incarceration declined between 2019 and 2024 and further investigation is needed to determine whether this was related to an increase in medications for opioid use disorder in county jails. A substantial proportion of the acute care population was recently incarcerated and is an important population for both payers and health systems to consider in efforts to reduce high cost care. ED visits can also serve as critical touchpoints for engaging this high‐risk, high‐need population. Additionally, for public health surveillance, ED visits can be a near‐real‐time indicator of population health trends in substance use‐related morbidity.

## 4 REVIEW OF STANDARDS‐BASED ELECTRONIC CASE REPORTING (eCR) FOR PUBLIC HEALTH SURVEILLANCE

### 4.1 Chanhee Kim, MPH, BSN, CIC, PHIT^1^; Larry Chen, BS^2^; Jacqueline Cassman, MPH^3^; Aasa Dahlberg Schmit, BSc^4^; Sarah Solarz, MPH^3^; Sripriya Rajamani, MBBS, PhD, MPH, FAMIA^1,5^

#### 4.1.1 ^1^School of Nursing, University of Minnesota, Minneapolis, Minnesota, USA; ^2^School of Medicine, Tufts University, Boston, Massachusetts, USA; ^3^Minnesota Department of Health, St. Paul, Minnesota, USA; ^4^HLN Consulting LLC, Mission Viejo, California, USA; ^5^Institute for Health Informatics, University of Minnesota, Minneapolis, Minnesota, USA

**Corresponding Author:** Sripriya Rajamani, sripriya@umn.edu

**Introduction:** Efficient surveillance of notifiable infectious diseases is critical for public health, yet traditional reporting methods (phone/fax/paper) are delayed, incomplete and inefficient. Electronic Case Reporting (eCR), built on HL7 standards, automates case reporting from healthcare to public health, enhancing timeliness, accuracy, and scalability of disease surveillance. This study assesses the impact of eCR on key metrics: timeliness, completeness, and reporting volume. Evaluation of informatics tools is a much‐needed step towards learning health systems.

**Methods:** A systematic review of U.S.‐based studies was conducted to assess the effectiveness of eCR. Articles published between 2010 and 2024 were identified through databases (MEDLINE®, Embase). The review timeframe was selected based on 2010 federal policies that promoted electronic health records (EHRs) adoption and laid the groundwork for standards‐based eCR. Of the 300 studies identified, 31 screened for eligibility with a focus on eCR's impact on public health reporting. Quality was assessed using Quality Assessment with Diverse Studies (QuADS) tool, and key findings were synthesized to highlight eCR's advantages and limitations.

**Results:** Table 1 presents the eight studies that met the inclusion criteria. These studies highlight significant improvements in reporting timeliness, data completeness, and reporting volume with eCR adoption. Quality assessment with QuADS tool (*n* = 13 criteria) revealed that most studies demonstrated robust methodological rigor, particularly in sampling methods, data collection, and analytical approaches. Studies (*n* = 8) consistently demonstrated that eCR enhanced the timeliness of disease reporting by reducing delays through automation. Completeness was significantly improved as eCR systems ensured the inclusion of essential data elements, with notable success reported in chlamydia and COVID‐19 cases. Several studies also emphasized eCR's scalability, with increased volume of reported cases, particularly for high‐priority diseases such as sexually transmitted infections and COVID‐19. Notably, some investigations explored barriers to eCR adoption, such as technological and organizational challenges, and provided insights into potential solutions, including enhanced training and infrastructure investments.

**Conclusions:** eCR, grounded in the HL7 standard, represents a transformative advancement in infectious disease surveillance, addressing inefficiencies inherent in traditional methods. By automating case reporting and enhancing capacity, eCR enables public health systems to manage higher case volumes with greater efficiency and quality. Policymakers must invest in infrastructure, standards‐based reporting and workforce training to fully leverage the full potential of eCR. These research findings will lead to learnings and to improve reporting processes for public health surveillance and lay the groundwork for learning health systems in public health.

**Acknowledgements:** We are grateful for the support provided by the Pauline A. Vincent Chair in Public Health Nursing, School of Nursing, University of Minnesota.

**Table:** **TABLE 1**. Summary of key findings from included studies

Study (Year)	Purpose	Design	Data sources	Diseases	Outcome	Findings
Dixon et al. (2020)	To evaluate the effect of a prepopulated notifiable disease reporting system on reporting rates, completeness, and timeliness.	Controlled before‐and‐after trial	Indiana HIE	Chlamydia, Gonorrhea, Hepatitis B, Hepatitis C, etc.	Completeness, Timeliness	Prepopulated eCR forms improved reporting rates and completeness for some diseases but faced challenges in timeliness for certain conditions.
Knicely et al. (2024)	To describe the development, implementation, and expansion of eCR in the United States.	Descriptive analysis	Multiple U.S. public health systems	Not specified (general infectious diseases)	Not specified	Highlighted key factors and challenges in scaling eCR across public health systems in the U.S.
Mishra et al. (2019)	To pilot an eCR system for Chlamydia and Gonorrhea reporting, demonstrating its potential to streamline surveillance.	Pilot study	Alliance Chicago's EHR and laboratory data	Chlamydia, Gonorrhea	Completeness, Timeliness	Pilot findings demonstrated that eCR streamlined reporting and reduced manual workload while improving data quality.
Mishra et al. (2021)	To design and implement a modified automated case event reporting platform to enhance laboratory reporting with clinical data.	Design and implementation study	PACER platform integrated with FHIR‐based systems	Chlamydia	Completeness	PACER enhanced eCR by integrating clinical data, improving the completeness of reports for Chlamydia.
Mishra et al. (2023)	To implement and evaluate an eCR system for Chlamydia and Gonorrhea in Illinois clinics to improve reporting accuracy and timeliness.	Implementation and evaluation study	EHR systems in Illinois clinics	Chlamydia, Gonorrhea	Timeliness	Implementation led to faster and more accurate reporting of Chlamydia and Gonorrhea cases in Illinois clinics.
Whipple et al. (2019)	To develop an eCR process to improve the quality of STD surveillance data, reduce time spent on investigations.	Descriptive analysis	UDOH's public health surveillance system	Chlamydia trachomatis infection, Gonorrhea, HIV Infection (adult), Syphilis (reactor)	Completeness, Timeliness, Volume	18 additional data elements for STD case were automatically transmitted through the eCR. The time required for clinical data transmission was also significantly reduced.
Rajamani et al. (2022)	To assess the implementation and data quality of COVID‐19 eCR at the MDH.	Descriptive analysis	Minnesota Electronic Disease Surveillance System	COVID‐19	Completeness, Volume	The implementation of eCR improved reporting rates for COVID‐19 cases and data completeness for some demographic variables.
Todd et al. (2022)	To automate the case reporting of Chlamydia and Gonorrhea to public health authorities in Oregon clinics.	Descriptive analysis	Oregon public health surveillance system	Chlamydia, Gonorrhea	Completeness, Timeliness	Automated reporting reduced errors and improved the timeliness of case reporting for Chlamydia and Gonorrhea in Oregon.

eCR: electronic Case Reporting; EHR: Electronic Health Record; FHIR: Fast Healthcare Interoperability Resources; HIE: Health Information Exchange; MDH: Minnesota Department of Health; STD: Sexually Transmitted Disease; PACER: Public Health Automated Case Event Reporting; UDOH: Utah Department of Health

## 5 MAKING DATA USABLE: MINNESOTA DEPARTMENT OF HEALTH TRAUMA AND EMS DATA

### 5.1 Evan D. Christensen, BA^1^; Sarah Tannert‐Lerner, BA^2^; Matthew Loth, PhD^1^

#### 5.1.1 ^1^Center for Learning Health System Sciences, University of Minnesota, Minneapolis, MN USA; ^2^Minnesota Department of Health, St. Paul, MN USA

**Corresponding Author:** Evan Christensen, chri4762@umn.edu

**Introduction:** Studying the continuum of care between traumatic incidents, pre‐hospital treatment and transportation, and arrival at a trauma center is crucial in order to improve best practices. However, bringing EMS and trauma data together can be a challenge due to duplicate entries, lack of standard variable format, and differing table structures. A collaboration between the Center for Learning Health System Sciences (CLHSS), the Translational Center for Resuscitative Trauma Care (TCRTC), and the Minnesota Department of Health (MDH), has advanced a clean database combining EMS and trauma care touchpoints, and has begun research using the data.

**Methods:** CLHSS used R to clean and prepare MDH data, including standardizing variables into a consistent format. Deduplication was performed so only those patients/encounters with completely unique trauma and EMS identification numbers were included, resulting in easier‐to‐combine trauma and EMS tables. Using these tables, CLHSS summarized many key demographic, treatment, and timeline variables, including age, biological sex, race/ethnicity, hospital trauma level, and length of stay. Differences in data sources were resolved throughout the process, prioritizing the source with fewer missing values–typically the trauma table. A data dictionary was then generated with table and variable names, definitions, missingness, summary statistics, and source table data.

**Results:** The created table summary and comprehensive data dictionary clarify which research questions the data is about to support. Through this partnership, CLHSS will continue to adapt, clean and process the data to best meet the needs of TCRTC's evolving research questions. An important consideration in future work is ensuring that deduplication is performed carefully, ensuring that it doesn’t underrepresent patients that suffered multiple trauma events and/or underwent an EMS transfer from one trauma center to another. Future work should also collaborate with MDH to review inclusion/exclusion criteria for trauma and EMS data to help ensure completeness and accuracy. This process had demonstrated the need for continued partnership between CLHSS, TCRTC, and MDH, investigation into prior data transformation, and continual improvement efforts.

**Conclusions:** Key lessons have been learned about the methods that have been and will be used to prepare the database. Both trauma and EMS data include multiple tables, often more granular than a “one‐row‐per‐encounter” structure. These contain repeated measures, event‐level details, and procedural data such as medications, vitals, and diagnoses. While this level of detail enables deeper analysis, it also requires careful aggregation and linkage for research projects focused on the encounter or patient level. Each project will need tailored strategies to ensure aggregation does not result in loss of critical detail. A future challenge will be combining data from multiple events, namely those related to trauma center transfers resulting in patients with a single trauma ID and multiple EMS IDs. This work will allow future studies to better account for patients’ continuum of care and provide usable conclusions regarding trauma patterns and outcomes at a system level.

## 6 HARNESSING ELECTRONIC HEALTH RECORDS TO CAPTURE CRUCIAL CONTEXTUAL INFORMATION ON INDIVIDUALS' SOCIAL SUPPORT SYSTEMS

### 6.1 Allison M. Gustavson, PT, DPT, PhD^1,2,3^; Hannele Nicholson, CCC‐SLP, PhD^4^; Sarah Garrett, RN^5^; Carla Amundson, MPH^1^; Huai Cheng, MD^2,5^; Howard Fink, MD^1,2,5^; Emily Hudson, PhD^1^; Edward Ratner, MD^2,5^; Erin Russel, DNP^6^; Hilary Mosher, MD^1,2,5^

#### 6.1.1 ^1^Veterans Affairs Health Services Research and Development Center for Care Delivery and Outcomes Research, Minneapolis Veterans Affairs Health Care System, Minneapolis, MN 55417 USA; ^2^Department of Medicine, University of Minnesota, Minneapolis MN 55455 USA; ^3^Department of Family Medicine and Community Health, Rehabilitation Science, University of Minnesota, Minneapolis, MN 55455 USA; ^4^Center for Veterans Research and Education, Minneapolis Veterans Affairs Health Care System, Minneapolis, MN, 55417 USA; ^5^Geriatric Research Education & Clinical Center, Minneapolis Veterans Affairs Health Care System, Minneapolis, MN, 55417 USA; ^6^University of Minnesota, School of Nursing, Minneapolis, MN 55455 USA

**Corresponding Author:** Allison M. Gustavson, PT, DPT, PhD, allison.gustavson@va.gov

**Introduction:** Formal and informal care partners are crucial for many older adults, yet information detailing their roles and relationships is not systematically documented in medical records. Electronic Health Records (EHRs) enable Learning Health Systems (LHS) to support outcomes for older adults through aggregating data. While formal and informal care partners are crucial for many older adults, information detailing care partner roles and relationships are not systematically documented in the Veterans Affairs (VA) EHR. As in most healthcare systems, the VA EHR tends to emphasize structured data and linear workflows, whereas care partner information is considered ‘soft data’ that are sensitive, unstructured, non‐linear, and distributed. Thus, a full picture of a Veteran's support community that evolves unpredictably over time is at odds with EHR infrastructure and workflows. Recognizing this concern, we sought to create a documentation template to structure clinically useful information that can both feed into an LHS cycle and serve the primary purpose of effective asynchronous communication between and across clinical teams. To do this, we convened an interdisciplinary team to develop a documentation solution with a resulting EHR note template. The purpose of this quality improvement project was to assess and iteratively refine this intervention and implementation effort in the context and with the lens of a LHS approach.

**Methods:** The design was a single‐site, rapid cycle quality improvement project guided by the Plan‐Do‐Study‐Act (PDSA) cycle and the Reach, Effectiveness, Adoption, Implementation, and Maintenance (RE‐AIM) framework. The note titled, “Current Veteran Support Team” (CVST), with structured and unstructured prompts, was developed and is currently live within the Minneapolis VA EHR CPRS (Computerized Patient Record System). The iterative process of developing the note was informed by interdisciplinary key informant interviews and the project team's knowledge of both clinical workflow and EHR data elements. First, clinical champions of various disciplines (e.g., social work, primary care, and rehabilitation) were alerted to its availability and offered one‐time training. To promote uptake of the note, we utilized a low‐cost, low resource strategy involving outreach to clinical teams followed by synchronous and asynchronous education to identify early adopters.

**Results:** Seven diverse VA clinical teams attended synchronous training on the CVST note and 15 notes were completed by providers over 6 months, with the majority being completed by Registered Nurses or Social Workers.

**Conclusions:** Uptake of the note by clinical team members has been slow, despite the importance of this information and exposure to training on use of the note. Entry of care partner information into the EHR can be conceptualized as both everybody's job and nobody's job. In other words, documenting care partner information does not fit obviously into an assigned workflow. A self‐complete version from the Veteran and/or care partner may provide a solution to overcoming barriers to obtaining this information. Future research is needed to evaluate how contextual information related to a Veteran's support network can be most effectively leveraged in clinical care and communicated through the EHR platform for potential impact on quality of care and patient, care partner, and system outcomes.

**Acknowledgements:** Support for this work was provided by the VA Office of Geriatrics and Extended Care. Dr. Gustavson's time is supported by the Minneapolis Veterans Affairs Center of Innovation, Center for Care Delivery and Outcomes Research (CIN 13‐406). The views expressed in this article are those of the authors and do not necessarily reflect the position or policy of the Department of Veterans Affairs or the United States Government.

## 7 FROM NOISE TO INSIGHT: LEVERAGING DATA TO DESIGN BETTER CLINICAL SYSTEMS AND MITIGATE ALARM FATIGUE

### 7.1 Benjamin Millmann, MSN, RN^1^; Casey Dollison, MSN, RN^1^.

#### 7.1.1 ^1^Clinical Technologies, Fairview Health Services, Minneapolis, MN, USA

**Corresponding Author:** Benjamin Millmann, benjamin.millmann@fairview.org

**Introduction:** Reducing alarm fatigue in healthcare is crucial for enhancing patient safety and clinician satisfaction, yet little research exists around best practices and standards for managing alarms, often resulting in highly variable organizational alarm configurations and staff experiences. Adding to the complexity of this problem are the extremely variable and ever‐changing capabilities of alarming systems, frequently evolving integration capabilities, and the obvious challenge of trying to push alarm standardization amongst different clinical specialties and care areas.

**Methods:** By using data‐driven analysis, rather than arbitrary or anecdotal‐based assessments of alarm fatigue, we were able to better evaluate and quantify alarm outliers, system pain‐points, and help drive standardization opportunities. To achieve this goal, our organization first established access to reliable data and reporting tools, and then worked to develop clear, overarching alarm management goals and strategies. We used this data to guide technology integration options, such as notification to mobile devices, to ensure these enhanced capabilities helped focus alarm notifications rather than just amplify them. This allowed us to be better prepared to navigate the intricacies of system‐specific alarm settings and design decisions. Finally, we created a system strategy that supported simplicity and standardization where possible but stopped short of creating a “one‐size fits all” package, which was vital for consistency and scalability.

**Results:** While following this approach resulted in clear opportunities to reduce alarm fatigue and overall alarm volumes through educated and improved alarm setting decisions, ensuring a consistent design across all care areas—while still accommodating clinical specialty independence and autonomy—proved quite difficult. Creating a strong, nurse‐led governance structure for making system‐wide alarm management decisions proved essential to our organization and helped manage the difficult decisions between population‐specific requests and the larger system design needs. Furthermore, clearly associating these alarm decisions to improvements in patient safety was far more challenging, and required substantial and dedicated resources to continuously track, compare, and evaluate change efforts to any clinical impacts.

**Conclusions:** By learning from the experiences of other healthcare institutions, creating a clear organizational governance structure for alarm management, and by employing data‐driven strategies to select and evaluate alarm decisions, hospitals can significantly decrease their alarm fatigue, improve patient safety, and enhance clinician responsiveness to critical alerts.

## 8 A LEARNING HEALTH SYSTEM APPROACH TO IMPROVING SYSTEM DELIVERY AND EVALUATION OF HIP SURVEILLANCE GUIDELINE ADHERENCE IN CHILDREN WITH CEREBRAL PALSY

### 8.1 Michael Peterson, MA^1^; Emily Andrisevic, MD^1^; Krystina Hopkins, MPH^1^; Kelzee Tibbetts MPH^1^; Meghan Munger, PhD, MPH^1^

#### 8.1.1 ^1^Gillette Children's Specialty Healthcare, Saint Paul, MN, USA

**Corresponding Author:** Michael Peterson, michaeljpeterson@gillettechildrens.com

**Introduction:** Cerebral palsy (CP) is the most common neuromotor condition in children, and hip dislocation affects up to 35%. Dislocation can cause pain, reduced mobility, lower quality of life, and lead to surgery [1]. Hip surveillance (HS) outlines a series of exams and hip x‐rays to detect hip displacement early, enabling timely and less invasive interventions to prevent dislocation. Despite evidence of effectiveness, fidelity is difficult to measure and suboptimal, limiting the health impact of HS [2].

We redesigned our HS program using implementation science, focusing on data infrastructure and medical provider behavior change to improve organizational fidelity to HS.

**Methods:** We performed a needs assessment, finding substantial provider performance and data gaps at our specialty pediatric hospital. Half of the children with CP were missing key functional status information to determine HS eligibility. In 2024, 39% of *known* eligible children were non‐adherent with HS. We targeted two provider behaviors: ordering hip x‐rays when due and entering functional information.

We used the Behavior Change Wheel (BCW), a planning framework to develop evidence‐based interventions to change behaviors. It is based on the Capability Opportunity Motivation for Behavior (COM‐B) model to understand what drives behavior [3, 4]. Our aims were to:

- **Identify provider determinants of adherence to HS**: We reviewed literature and interviewed 12 Gillette staff (6 providers, 6 nurses). Determinants were targets for implementation and monitored through a theory‐consistent survey that was sent to providers involved in HS[5].
- **Design a comprehensive implementation package**: We used the BCW framework to select evidence‐based interventions to change identified determinants and target behavior. An interdisciplinary workgroup reached consensus on feasible and impactful intervention components using BCW's Affordability, Practicality, Effectiveness, Acceptability, Side‐effects, Equity (APEASE) criteria.

**Results:** Assessment of determinants showed consistent results between interviews and baseline survey responses (*N* = 21). Using the COM‐B model, we found psychological capability, reflective motivation, and physical and social capability must change to improve HS guideline adherence.

Corresponding interventions functions included:

- Persuasion
- Education
- Training
- Environmental restructuring
- Enablement

We then selected behavior change techniques aligned with intervention functions and grouped them into implementation strategies to specify implementation activities to support behavior change (Figure 1):

- Electronic medical record modifications (e.g., reminder system),
- Education and persuasive messaging
- Training
- Provider section goal setting/planning

**Figure:**
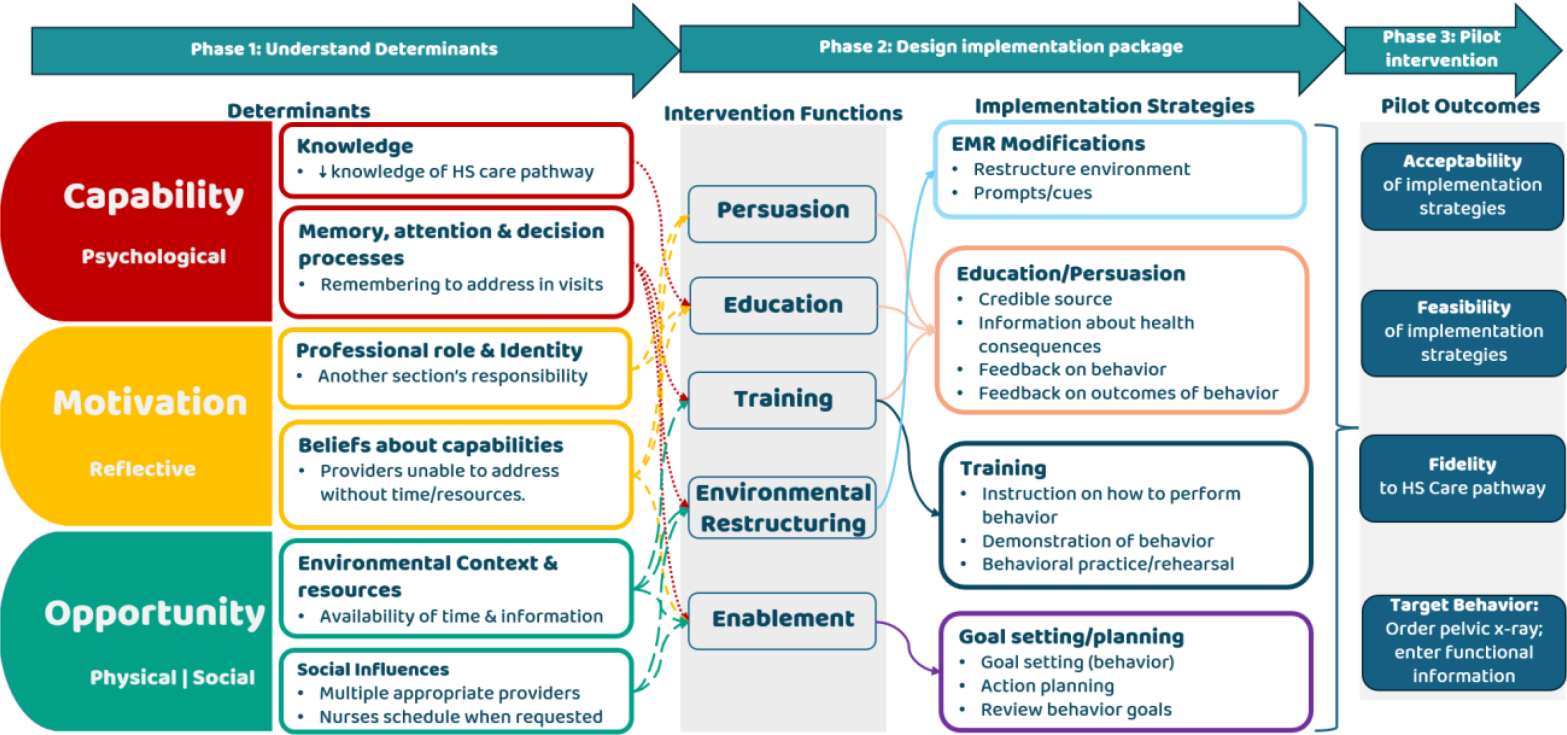
**Figure 1:** Conceptual diagram of hip surveillance implementation plan from BCW planning process:

**Conclusions:** Meaningful clinician engagement combined with a BCW planning framework led to identification of evidence‐based implementation strategies tailored to provider perspectives that were previously unconsidered. Our next step is to evaluate the impact of the implementation package on early implementation outcomes [6] and determinants, utilizing the BCW process in future improvement cycles.

A limitation of our project was not including patient and family perspectives. While provider determinants to guideline‐concordant care are important, inclusion of lived experience in our planning may open new opportunities to support adherence to HS. To inform and improve the reach of this evidence‐based intervention, we will partner with a new CP Family Council to incorporate patient and family perspectives on HS participation.

**Acknowledgements:** The authors would like to thank colleagues in the CPRN for their collaboration in improving HS across participating institutions.

**References**

1. Miller, S.D., et al., *Prevention of hip displacement in children with cerebral palsy: a systematic review*. Dev Med Child Neurol, 2017. 59(11): p. 1130–1138.

2. Willoughby, K.L., et al., *Health professionals' experiences and barriers encountered when implementing hip surveillance for children with cerebral palsy*. J Paediatr Child Health, 2019. 55(1): p. 32–41.

3. Michie, S., L. Atkins, and R. West, *The Behaviour Change Wheel: A Guide to Designing Interventions*. 2014: Silverback Publishing.

4. Atkins, L., et al., *A guide to using the Theoretical Domains Framework of behaviour change to investigate implementation problems*. Implementation Science, 2017. 12(1): p. 77.

5. Huijg, J.M., et al., *Measuring determinants of implementation behavior: psychometric properties of a questionnaire based on the theoretical domains framework*. Implementation Science, 2014. 9(1): p. 33.

6. Proctor, E., et al., *Outcomes for implementation research: conceptual distinctions, measurement challenges, and research agenda*. Adm Policy Ment Health, 2011. 38(2): p. 65–76.

## 9 DECREASING RACIAL DISPARITIES IN OUTPATIENT CARDIAC REHABILITATION REFERRALS FOR PATIENTS WITH AN ACUTE MYOCARDIAL INFARCTION

### 9.1 Whitney Quast, MS, CEP, CCRP, CSSBB^1^; Aaron Pergolski, MA, CEP, CCRP^1^; David Pelletier, BIE, MMSE, LSSBB, CLM, CPPM^1^; Tammy Lampro, MT, MBA, CQE, CSSBB^1,2^; Jeremy Van’t Hof, MD, MS^2^; & Austin Hoeg, MD, MPH^3^

#### 9.1.1 ^1^Fairview Health Services, Minneapolis, MN, USA; ^2^University of Minnesota Physicians, Minneapolis, MN, USA; ^3^University of Minnesota School of Medicine, Minneapolis, MN, USA

**Corresponding Author:** Whitney Quast, whitney.quast@fairview.org

**Introduction:** Racial disparities continue to affect healthcare outcomes for Black, Indigenous, and People of Color (BIPOC), including in cardiac care after acute myocardial infarction (AMI). Referral to a cardiac rehab outpatient program is a Class I indication in clinically managing AMI after hospital discharge. At M Health Fairview, BIPOC adults were 7% less likely (*p* = 0.00) than white adults to receive referrals to outpatient cardiac rehabilitation programs, despite strong evidence that cardiac rehab reduces hospital readmissions and cardiac mortality.

**Methods:** A root cause analysis was conducted through a comprehensive protocol assessment with regional stakeholders (e.g., Cardiologists, Hospitalists, Clinical Directors, Clinical Supervisors) from M Health Fairview's University of Minnesota Medical Center, Southdale Hospital, Ridges Hospital, St. John's Hospital, Lakes Medical Center, Grand Itasca Clinic & Hospital, Woodwinds Hospital, and Range Medical Center. Common themes (i.e., length of stay, language barriers, delayed referral, no referral, ‘percutaneous intervention (PCI)’ order set) within the pre‐intervention state were identified and charted in a process map. Interventions included: (1) translating educational resources, (2) editing the ‘PCI’ order set from M Health Fairview's East Region to pre‐check outpatient cardiac rehabilitation, (3) adding cardiac rehab to medical providers’ orientation checklist, (4) establishing bimonthly inpatient cardiac rehabilitation collaboration, and (5) editing the ‘Discharge to Home’ order set to clarify indications for outpatient therapies to increase provider awareness of qualifying diagnoses. Outcomes of interest were AMI‐indicated outpatient cardiac rehab referral rates for M Health Fairview's BIPOC and white population. Access, the average number of days post‐discharge that patients began cardiac rehab, was also assessed as a balancing measure.

**Results:** In the analysis of the pre‐ and post‐intervention data, AMI‐indicated outpatient cardiac rehabilitation referral rates increased from 35% to 46% (*p* = 0.01) in BIPOC adults and 42% to 49% (*p* = 0.00) in white adults from March 2022 through June 2025 across the M Health Fairview System. There was no statistically significant difference (*p* > 0.46) in the referral rates of BIPOC compared to white patients in the last three measurement periods. Additionally, the average number of days before patients began cardiac rehab post‐discharge was not negatively impacted (See Figure 1 below).

**Conclusions:** Root cause analysis and targeted protocol changes led to significant improvements in cardiac rehab referral rates for BIPOC patients. These changes can improve health outcomes, reduce hospital readmissions, and enhance reimbursement through incentive payment models. The next phase will focus on improving enrollment and adherence rates, as well as measuring and addressing disparities within other cardiovascular diagnoses.

**Figure:**
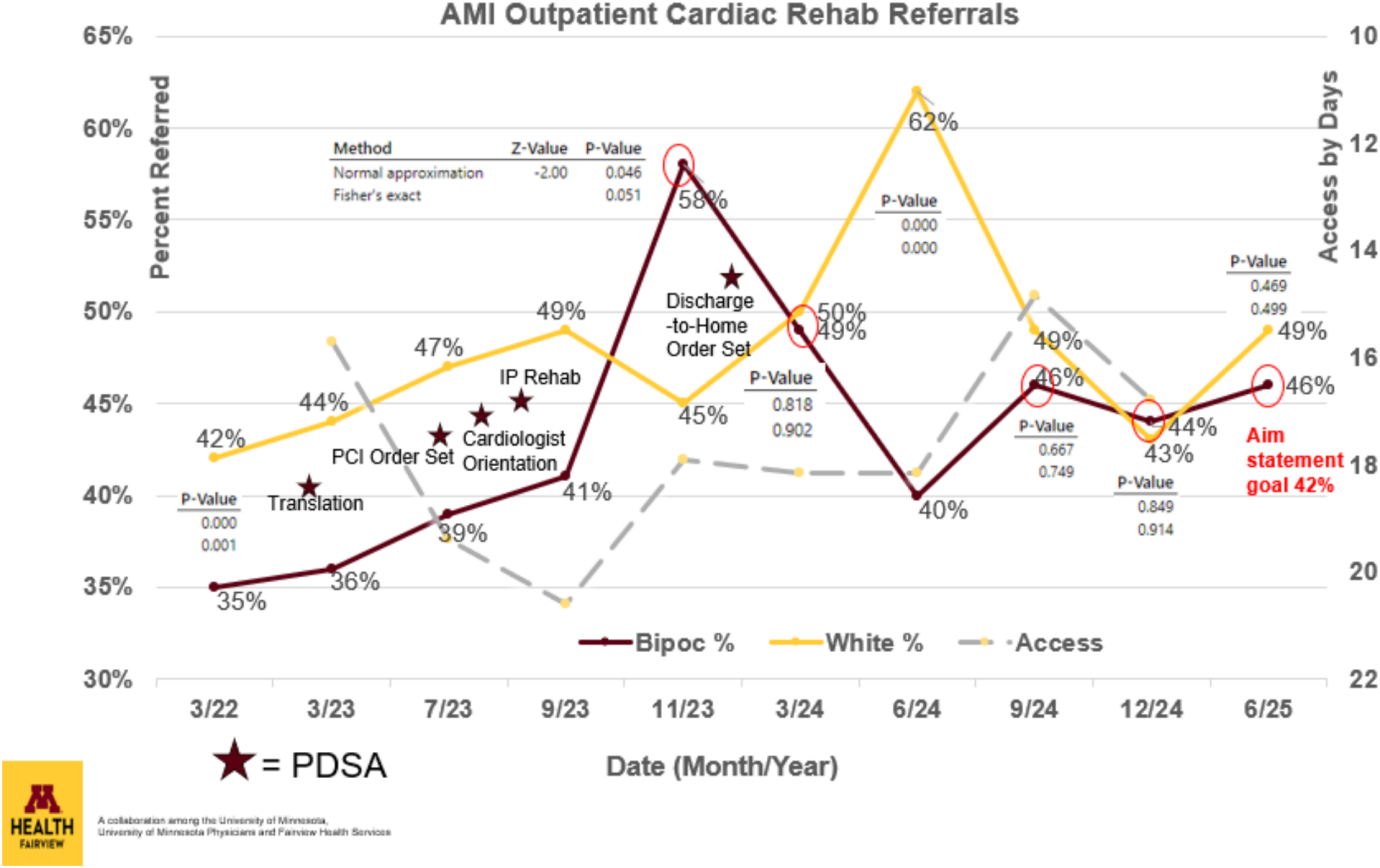
**Figure 1**.

**Acknowledgements:** The authors would like to thank the M Health Fairview Quality Improvement Collaborative.

## 10 EXPANDING THE BOUNDARIES OF LEARNING HEALTH SYSTEMS: 3 CASE EXAMPLES OF INTEGRATED PUBLIC HEALTH AND HEALTH CARE APPROACHES

### 10.1 Renée Kidney, PhD, MPH^1^; James Peacock, PhD, MPH^1^; Ally Fujii, MPH^1^; Chris Brueske^1^; Ann Zukoski, DrPH, MPH^1^

#### 10.1.1 ^1^Minnesota Department of Health, St. Paul, MN USA

**Corresponding Author:** Ann Zukoski, ann.zukoski@state.mn.us

**Introduction:** Minnesota has rich data resources and a broad community of public health and health care professionals committed to improving outcomes through data‐driven approaches who bring diverse perspectives to this work. Despite their shared mission, work often occurs in siloes and tends to be reactive. Ongoing statewide data modernization initiatives present new opportunities for collaboration among data scientists, informaticians, clinicians, and public health professionals, alongside community partners. This project describes how the Minnesota Department of Health (MDH) is building a collaborative vision of a bridged health and public health learning system for chronic disease. We highlight three case examples that demonstrate how Learning Health System (LHS) principles can be expanded to include public health partners and a broader population health lens.

**Methods:** MDH identified three case examples that demonstrate the potential for bridging public health and clinical thinking to improve population health and data modernization in Minnesota. These case examples focus on: (1) statewide stroke data and learning collaboratives; (2) cardiac rehabilitation (CR) access, quality, and equity; and (3) caregiver health and wellbeing using multiple data sources.

Each pilot involved partnership with health systems or statewide agencies, use of existing data infrastructure, and intentional engagement of stakeholders to develop or refine tools, dashboards, or systems‐level approaches to improving chronic disease outcomes. Public health and clinical stakeholders jointly analyzed data, identified gaps, and co‐designed strategies to strengthen systems‐level learning.

**Results: *Stroke System Learning:*** MDH maintains a clinical registry of acute stroke patients for 121 Minnesota Stroke System hospitals, capturing almost all stroke hospitalizations. Through real‐time performance measure monitoring, analysis, and participation in learning collaboratives, hospitals can address stroke care inequities. This approach enhances clinical system learning with co‐designed quality improvement initiatives, with a clear role for public health to support hospitals as they address and evaluate systems‐level changes to improve patient outcomes.

***Cardiac Rehabilitation (CR):*** Fragmented care makes it difficult for CR professionals to measure enrollment, participation, and completion, especially when patients cross health system boundaries. MDH's published the first public CR dashboard with statewide data, which provides visibility into inequities and gaps, empowering CR programs to set practical and aspirational goals. Impacts include reduced patient parking fees, closing equity gaps in automatic referrals, and identifying ways to reach eligible patients who never enroll in CR.

***Caregiver Health:*** The Minnesota Board on Aging's Committing to Caregiving workgroup adopted a systems‐level public health approach to understand caregiver needs. MDH supported the inclusion of state‐level BRFSS data about caregiver demographics, health, and wellbeing and aligned it with data from MBA‐led programs. This approach supports a broader understanding of care givers health needs.

**Conclusions:** Expanding LHS work to intentionally include public health and population‐level perspectives is critical for reducing health inequities in Minnesota. This approach strengthens upstream prevention, identifies gaps in traditional data models, expands focus beyond individuals already engaged in care, and reveals how fragmented systems impact outcomes. Data modernization efforts make meaningful collaboration increasingly possible. Real‐time, system‐wide data is essential for addressing inequities at a structural level. A combined public health and clinical learning system offers a powerful model for improving outcomes in new and coordinated ways.

## 11 EXAMINING EHR‐BASED PREVALENCE ESTIMATES AND LOCAL PUBLIC HEALTH SURVEY DATA: RESULTS FROM HEALTH TRENDS ACROSS COMMUNITIES (HTAC) AND THE SURVEY OF THE HEALTH OF ALL THE POPULATION

### 11.1 David Johnson, MPH^1^; Abby Jessen, MPH^1^; Komal Mehrotra, MHS, MS^1^; Urban Landreman, MS, MBA^1^; Mei Ding, MD, MS^1^; Renee Van Siclen, MPP^2^; Peter Bodurtha, MPP^2^; Paul Drawz, MD, MHS, MS^3^; Tyler Winkelman, MD, MSc^2^

#### 11.1.1 ^1^Hennepin County Public Health, Minneapolis, MN, USA; ^2^Hennepin Healthcare Research Institute, Minneapolis, MN USA; ^3^Department of Medicine, University of Minnesota, Minneapolis, MN USA

**Corresponding Author:** Dave Johnson, david.johnson2@hennepin.us

**Introduction:** The Survey of the Health of All the Population and Environment (SHAPE) is an adult health survey conducted every four years by Hennepin County Public Health in Minnesota. SHAPE collects data on overall population health, including mental health, access to healthcare, health behaviors, substance use, and neighborhood factors.

For the first time, public health agencies and health systems in Minnesota have access to summary prevalence data derived from clinical encounters across the state's 11 largest healthcare systems through the Health Trends Across Communities (HTAC) initiative. HTAC, a project of the Minnesota EHR Consortium (MNEHRC), uses a distributed data model to produce timely electronic health record (EHR)–based prevalence estimates. This study examines and compares prevalence estimates for chronic disease, mental health, and substance use between HTAC and the 2022 SHAPE survey.

**Methods:** We selected four health indicators available in both HTAC and SHAPE: diabetes, hypertension/high blood pressure, depression, and alcohol use. HTAC estimates reflect Minnesota residents who received care at a participating health system in the last three years and had a diagnosis for one of the indicators in the last five years. Nearly all Hennepin County residents are represented in HTAC data. SHAPE estimates were generated from a random address‐based sampling design that utilized a mixed‐mode mailed and in‐person data collection strategy. SHAPE excludes institutionalized populations and those in military or correctional facilities. In 2022, the response rate was 22.6%, with 8591 respondents. For this analysis, both data sources were limited to Hennepin County adults (18+). Prevalence estimates were stratified by race and ethnicity, sex, and age group (e.g., 18–24, 25–44). Data were analyzed geographically at the county and sub‐county level, with sub‐county geographies based on the 10 reporting areas used in SHAPE which are aggregations of census tracts.

**Results:** Diabetes prevalence estimates were concordant across data sources: 8.0% in HTAC and 5.9% in SHAPE. For hypertension, HTAC estimated a prevalence of 26% in adults, compared with 16.2% in SHAPE. Differences for alcohol use in the data sets were substantial. HTAC estimate that, overall in Hennepin County, 4% of adults had a diagnosis related to alcohol use disorder. The SHAPE 2022 survey reports excessive alcohol use at a much higher rate, at 59.6%. This is reflective of differences in clinical diagnostic versus self‐reported behavioral approaches to measurement of alcohol use.

**Conclusion:** Comparing self‐report survey data with EHR prevalence data allows local public health departments to identify potential ways to streamline survey processes and data collection efforts. HTAC represents a significant advancement in public health monitoring by providing timely and granular diagnosis‐based data. The SHAPE survey remains essential for capturing vital social determinants and population characteristics that are not available in EHRs. Both SHAPE and HTAC provide detailed data on the health of Hennepin County residents. Differences in methodology and approach are important to recognize and help define the advantages and limitations of these respective approaches to measuring population health prevalence.

## 12 MN‐AIR (MINNESOTA AI AND INFORMATICS RESOURCE) HUB: ASSESSING STATEWIDE AI NEEDS WITH HEALTH IT LEADERSHIP

### 12.1 Paige Nong, PhD^1^; Anna Schulte, MPH; Chloe Botsford, MPH^2^; Krystal Hosch, MPH^2^; Genevieve B. Melton, MD, PhD^2,3,4^

#### 12.1.1 ^1^School of Public Health, University of Minnesota, Twin Cities, Minneapolis, MN, USA; ^2^Center for Learning Health System Sciences, University of Minnesota, Twin Cities, Minneapolis, MN, USA; ^3^Department of Surgery, University of Minnesota, Twin Cities, Minneapolis, MN USA; ^4^Institute for Health Informatics, University of Minnesota, Twin Cities, Minneapolis, MN USA

**Corresponding Author:** Paige Nong, PhD, nong0016@umn.edu

**Introduction:** Health systems are increasingly adopting artificial intelligence (AI) tools for clinical care and operations. However, governance, vendor transparency, and resource allocation remain significant challenges. To address these issues, the Minnesota AI and Informatics Resource (MN‐AIR) Hub was established to produce rigorous empirical research and compile valuable resources that support safe and effective AI use across heterogeneous healthcare delivery organizations.

***Objective:*** (1) To capture perspectives from health system leaders across the state of Minnesota on current AI use, governance strategies, primary challenges, and resource needs, and (2) to inform empirical research that will bolster high‐value digital transformation.

**Methods:** An in‐person facilitated session was held during the September 2025 Advances in Learning Health System Sciences (AiLHSS) conference with ten health system leaders representing six health systems from across the state of Minnesota. Participants were in leadership positions related to health information technology (IT), AI, and data analytics. The meeting was recorded, and a qualitative researcher took detailed notes. The research team facilitated discussion across key areas informed by the literature: AI implementation status and maturity, barriers to effective governance and use, and best practices or resources to support safe, effective AI use. Participants engaged in structured brainstorming and facilitated discussion across these domains, identifying shared challenges and desired solutions. Themes were identified and compiled to guide next steps for empirical research and resource sharing.

**Results:** Participants reported active use of multiple AI tools, primarily for documentation and operations. Clinical use cases were rare. Participants’ governance strategies included broad stakeholder engagement (e.g., cybersecurity and legal) and vendor vetting, though vendor‐provided information was often insufficient to make informed decisions about specific AI tools. Shared concerns included scoping AI governance due to the rapid expansion of AI across IT tools, standardizing evaluation practices, monitoring tool effectiveness, and preventing clinician de‐skilling. Participants reported the resources they currently use and find valuable; federal guidelines (Food and Drug Administration Software as a Medical Device), the National Institute of Standards and Technology AI Risk Management Framework, Coalition for Health AI model cards, and electronic health record vendor resources. Desired resources included actionable governance guidelines, education for staff, dashboards for model performance and return on investment, and tools that facilitate vendor transparency.

**Conclusions:** Health system leaders expressed strong interest in collaboration to support safe and effective AI use and overcome shared barriers. MN‐AIR prioritizes empirical evidence and sharing valuable resources identified by participants, including best practices and educational materials. A dedicated website will host these resources. Continued engagement will focus on ensuring safety and effectiveness, evidence‐based decisions, vendor accountability, and peer learning.

## 13 IDENTIFYING OPIOID USE DISORDER ARCHETYPES AMONG HENNEPIN HEALTHCARE PATIENTS USING CLUSTERING ANALYSIS, 2020–2024

### 13.1 Renee Van Siclen, MPP^1^; Peter Bodurtha, MPP^1^; Tyler Winkelman, MD, MSc^1^

#### 13.1.1 ^1^Hennepin Healthcare Research Institute, Minneapolis, MN USA

Corresponding Author: Renee Van Siclen, rvansiclen@hhrinstitute.org

**Introduction:** Opioid use disorder (OUD) affects nearly 65,000 people across Minnesota, impacting communities across demographic and socioeconomic lines. This makes a one‐size‐fits‐all approach to OUD prevention and treatment insufficient. While prior research has documented demographic differences among groups of people with OUD, fewer studies have examined how differences in health status and healthcare use can inform treatment in a clinical setting. This study uses clustering analysis to identify OUD patient archetypes among Hennepin Healthcare patients, grouping individuals based on patterns in health conditions, healthcare use, and interactions with other systems like criminal justice and homelessness services. By identifying clinically meaningful clusters, this work aims to support more individualized, needs‐based approaches to OUD treatment. The goal of this work is to design OUD treatment that best meets the needs of patients.

**Methods:** Electronic health record (EHR) data were collected from 38,000 Hennepin Healthcare patients from 2020 to 2024. The analytic sample was limited to individuals with at least one healthcare encounter in the past three years and an OUD diagnosis in the previous five years. Using dimensionality reduction followed by latent class analysis,patients were grouped according to variables reflecting:

- **Healthcare utilization** (e.g., number of outpatient visits)
- **Co‐occurring health conditions**, including physical and behavioral health and substance use
- **Interactions with other systems**, including experiences of homelessness or incarceration during the study period
- **Prescription history**, including short‐ and long‐term opioid prescriptions and use of medications for opioid use disorder (MOUD)

Demographic variables were intentionally excluded from the clustering algorithm to avoid potential age, race, or gender bias in the formation of clusters. Demographic summaries were created for each of the groups following clustering.

**Results:** Preliminary analysis suggest four distinct OUD archetypes, including (a) younger patients with low overall healthcare utilization (40%–45% of sample); (b) young adults who rely primarily on emergency department services and frequently experience housing instability (25%–30%); (c) intensive healthcare users with complex medical, behavioral health, and social needs (5%–10%); and (d) older adults whose opioid use primarily relates to pain management, representing approximately 20%–25% of the sample.

The early findings support the hypothesis that patients with OUD represent heterogeneous subgroups with varying circumstances, needs, and patterns of service use.

**Conclusions:** The findings indicate that there is clinically meaningful heterogeneity among patients with OUD which could inform the creation of different care pathways based on patient characteristics and needs. For instance, a young adult using intravenous drugs and primarily interacting with the ED may require more immediate approaches to harm reduction, while someone with a more established relationship to their primary care provider may benefit from ongoing medication management. Implementing a more tailored approach based on a patient's health needs and relationship with the healthcare system could improve the effectiveness of treatment strategies and support a more comprehensive approach to substance use treatment. Future research could include expanding the analysis to other health care systems in order to determine whether these archetypes hold true on a larger scale or to adapt these archetypes to encompass a broader population.

**Data Availability Statement:** Data sharing not applicable to this article as no datasets were generated or analysed during the current study.

